# Mast cell activation is characterized by upregulation of a functional anaphylatoxin C5a receptor

**DOI:** 10.1186/1471-2172-9-29

**Published:** 2008-06-17

**Authors:** Afsaneh Soruri, Jasmin Grigat, Ziba Kiafard, Jörg Zwirner

**Affiliations:** 1 Department of Cellular and Molecular Immunology, Georg-August-University Göttingen, Humboldtallee 34, D-37073 Göttingen, Germany

## Abstract

**Background:**

Mast cells (MC) are key effector cells of allergic diseases and resistance to helminthic parasites and induce or amplify diverse innate and adaptive immune responses. The signals controlling MC mobilization during inflammation are not fully understood.

**Results:**

Since anaphylatoxins are attractive candidates as MC chemoattractants, we investigated expression and function of anaphylatoxin receptors in murine MC. Precursor cell-derived MC cultured with IL-3 in the presence or absence of SCF did not express significant amounts of surface C5a receptor (C5aR) or C3a receptor (C3aR). MC required approximately 4 h of stimulation with Ag (DNP-albumin, following preincubation with IgE anti-DNP), ionomycin, or PMA to enable a strong chemotactic response towards C5a, paralleled by a distinct C5aR upregulation. Likewise, C5a induced intracellular calcium fluxes solely in activated MC. In contrast, C3a proved to be a weak MC chemotaxin and unable to increase intracellular calcium. Primary peritoneal MC did not express detectable amounts of anaphylatoxin receptors, however, similar to precursor cell-derived MC, stimulation with Ag or ionomycin for 4 h induced a prominent surface expression of C5aR whereas C3aR remained undetectable.

**Conclusion:**

Collectively, our results suggest that Ag-dependent as well as -independent activation induces an inflammatory MC phenotype which is distinguished by neoexpression of a functional C5aR as a novel effector mechanism in MC-mediated pathogenesis.

## Background

Many forms of infection or tissue injury lead to activation of the complement system resulting in the cleavage of complement components C3 and C5 and generation of the anaphylatoxins C3a and C5a [1]. Anaphylatoxins are responsible for recruiting and activating leukocytes, particularly phagocytic cells such as granulocytes and monocytes/macrophages and are involved in inflammatory, autoimmune and allergic diseases [2-4]. Anaphylatoxins perform their functions by engaging specific receptors which are closely related members of the rhodopsin family of seven transmembrane-spanning G protein-linked receptors.

MC have long been described as effectors of IgE-dependent immuneresponses that mediate immediate hypersensitivity reactions associated with allergic phenomena and host resistance to helminthic parasites, and are now also implicated in different autoimmune and inflammatory disease models [5,6].

The signals controlling MC recruitment and migration within tissues are poorly understood, but anaphylatoxins are particularly attractive candidates as MC chemoattractants during inflammation. In humans, for example, skin-derived MC have been shown to be sensitive to C5a and C3a whereas MC from the lung were not [7-10]. Studies with the immature human mast cell line HMC-1 even suggested C3a to be one of the most effective mast cell chemoattractants [11,12]. Furthermore, anaphylatoxin receptor expression may depend on variations in the local microenvironment since synovial MC expressed C5aR exclusively in inflamed tissue of rheumatoid arthritis patients [13,14].

The understanding of the pathophysiological and biochemical basis of the differential expression of anaphylatoxin receptors on MC subtypes is hampered by our scarce, sometimes controversial knowledge on the expression of anaphylatoxin receptors in rodent MC. Whereas C5a was able to degranulate skin-derived murine MC, peritoneal MC were found to be unresponsive [15]. On the other hand, C5aR on peritoneal MC was observed to be instrumental in a mouse model of zymosan-mediated peritonitis [16] whereas rat peritoneal MC degranulated in response to C3a and C3a(desArg) by a receptor-independent mechanism [17]. Clearly, studies of the interactions between MC and anaphylatoxins are still in their infancy despite their well-appreciated roles in allergy, infection and autoimmunity.

The purpose of the present study was (1) to investigate the impact of different modes of MC activation on the expression and function of anaphylatoxin receptors, (2) to compare precursor cell-derived MC generated in vitro with primary MC purified from the peritoneal cavity, and (3) to uncover differences in the expression profiles of C5aR and C3aR.

## Results

### Anaphylatoxin receptors on in vitro generated MC

**Figure 1:**
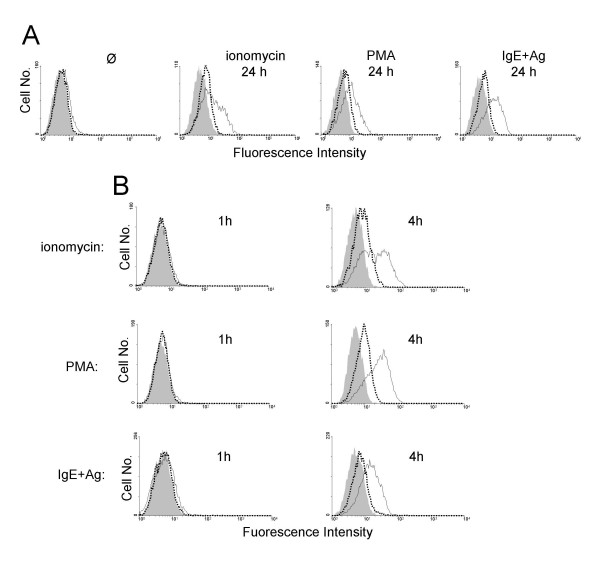
**Anaphylatoxin receptors are expressed on activated MC**. Precursor cell-derived murine MC cultured with IL-3 and SCF were treated or not (Ø) with ionomycin, PMA, or Ag (DNP-albumin, following a 24 h preincubation period with IgE anti-DNP) for 24 h (A) or for 1 h and 4 h, respectively (B). Subsequently, MC were stained by indirect immunofluorescence and analyzed by FACS. Filled histograms indicate staining with rat IgG1 control mAb, open histograms (solid lines) with mAb 1240 against murine C5aR, and open histograms (dotted lines) with mAb 1G4 against murine C3aR. One representative experiment each of at least 3 is shown.

Murine precursor cell-derived MC cultured in the presence of IL-3 and SCF were investigated for anaphylatoxin receptor expression using specific mAb against C5aR and C3aR, respectively. Anaphylatoxin receptor levels were found to be below the theshold of flow cytometric detection on resting MC. However, MC stimulation for 24 h with the calcium ionophore ionomycin, the protein kinase C activator PMA or Ag (DNP-albumin, following a 24 h preincubation period with IgE anti-DNP) resulted in a distinct increase in surface C5aR levels but only a weak C3aR upregulation (Fig. 1A). A time course study revealed that stimulation of MC for 1 h with ionomycin, PMA, or Ag was not sufficient to elevate anaphylatoxin receptor levels whereas 4 h of incubation resulted in a prominent expression of C5aR (Fig. 1B).

### C5a-induced MC functions

**Figure 2:**
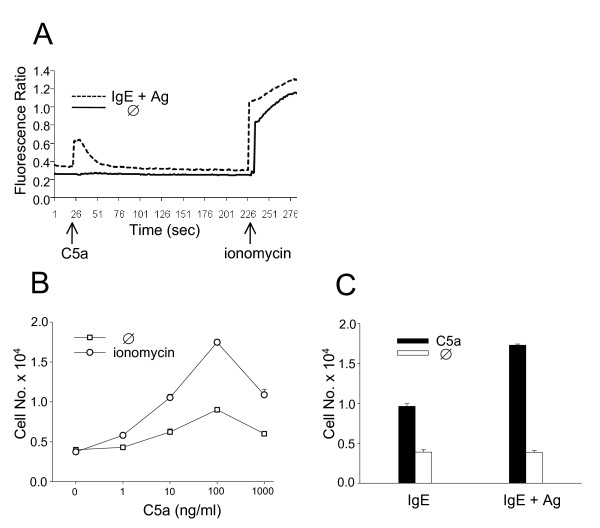
**Activated MC respond to C5a in vitro**. Precursor cell-derived murine MC cultured with IL-3 and SCF were treated or not (Ø) with Ag (DNP-albumin, following a 24 h preincubation period with IgE anti-DNP) for 4 h (A) or ionomycin for 24 h (B). In (C), MC were preincubated with IgE followed or not by Ag treatment for another 24 h. Subsequently, calcium fluxes in response to C5a (1 μg/ml) and ionomycin (750 ng/ml) (A) or in vitro chemotaxis towards C5a (different concentrations in B; 100 ng/ml in C) was measured. One representative experiment of 3 is shown in (A), mean values (± SEM) of 3 independent experiments each in (B, C).

In a next step, we looked for functional consequences of C5aR upregulation. C5a was unable to induce calcium fluxes in resting MC but, following stimulation with Ag (after IgE priming) for 4 h (Fig. 2A) or 24 h (data not shown), a distinct rise in intracellular calcium was observed. In line with this finding, stimulation with ionomycin (Fig. 2B) or Ag (following IgE priming) (Fig. 2C) augmented MC chemotaxis toward C5a in vitro.

**Figure 3:**
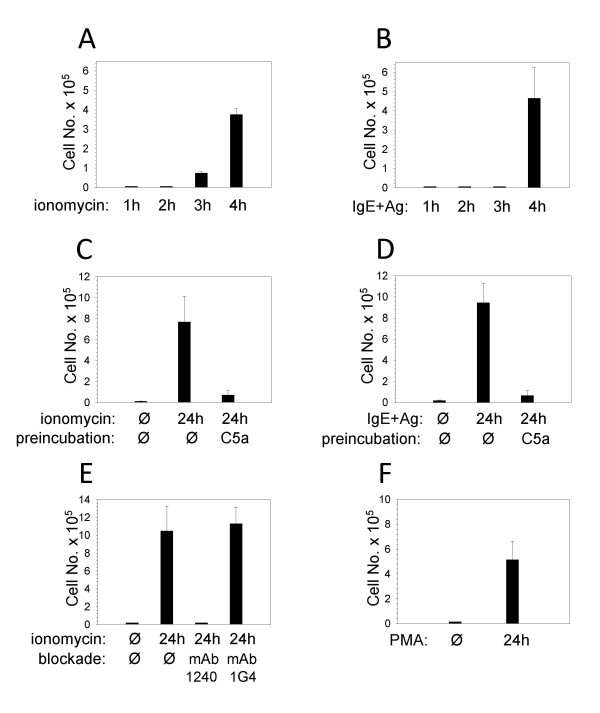
**Activated MC respond to C5a in vivo**. Precursor cell-derived murine MC cultured with IL-3 and SCF were treated or not (Ø) with ionomycin, PMA or Ag (DNP-albumin, following a 24 h preincubation period with IgE anti-DNP) for different periods as indicated. Subsequently, MC were labeled with PKH26. In (C, D), labeled MC were additionally preincubated at 37°C without (Ø) or with C5a (2 μg) to induce receptor desensitization. In (E), labeled MC were preincubated on ice without (Ø) or with mAb 1240 (20 μg, rat IgG1) to block C5aR, or with mAb 1G4 (20 μg, rat IgG1), as a control. Thereafter, labeled MC were injected i.v. into syngeneic BALB/c mice together with C5a (10 μg) i.p.. After 4 h (A, B, E) or 24 h (C, D, F), peritoneal cells were harvested and labeled migratory cells identified by FACS analysis. Mean values (± SEM) of 3 (A, B, D) or 4 (C, E, F) independent experiments each are shown.

Studying MC chemotaxis in an in vivo migration model, the distinction between C5a-induced mobilization of resting and activated MC was even more pronounced. PKH26-labeled MC were recruited into the peritoneal cavity by C5a injections exclusively after stimulation with ionomycin for at least 3 h (Fig. 3A) or with Ag (following IgE priming) for not less than 4 h (Fig. 3B).

Preincubation of activated MC with C5a abolished the subsequent chemotactic response to C5a in vivo, most likely as a result of receptor desensitization (Fig. 3C, D). Furthermore, anti-murine C5aR mAb 1240 abrogated in vivo migration of ionomycin-stimuled MC toward C5a (Fig. 3E) whereas anti-murine C3aR mAb 1G4 did not. These experiments confirm the receptor-specific nature of C5a-induced MC mobilization in vivo.

In parallel to the rise in C5aR expression on the cell surface (Fig. 1A), PMA-stimulated MC were also mobilized by C5a in vivo (Fig. 3F).

### C3a-induced MC functions

**Figure 4:**
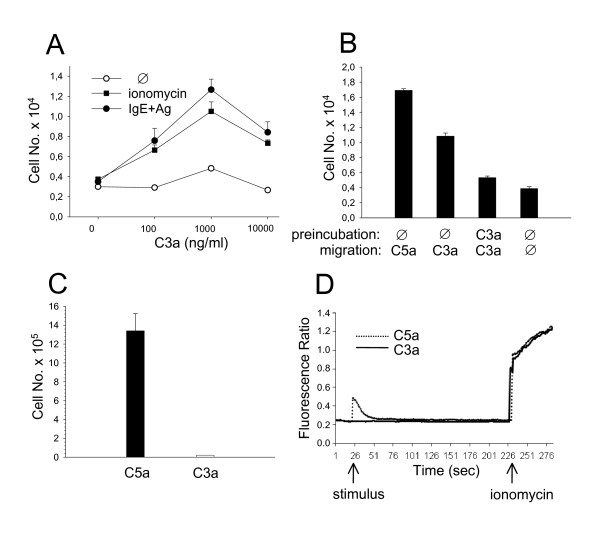
**C3a is a weak MC chemotaxin**. Precursor cell-derived murine MC cultured with IL-3 and SCF were treated or not (Ø) with ionomycin (A-C) or Ag (DNP-albumin, following a 24 h preincubation period with IgE anti-DNP) (A, D) for 24 h. Subsequently, in vitro chemotaxis towards different concentrations of C3a (A) or optimal concentrations of C5a (100 ng/ml) and C3a (1000 ng/ml) (B) was measured. In (B), MC were additionally preincubated at 37°C without (Ø) or with C3a to induce receptor desensitization. In (C), MC were labeled with PKH26 and injected i.v. into syngeneic BALB/c mice together with C5a (10 μg) or C3a (50 μg) i.p.. 24 h later, peritoneal cells were harvested and labeled migratory cells identified by FACS analysis. In (D), calcium fluxes in response to C3a (10 μg/ml) or C5a (1 μg/ml) and ionomycin (750 ng/ml) were measured. Mean values (± SEM) of 3 independent experiments each (A-C) and 1 representative experiment of 3 (D) are shown.

After establishing the correlation between MC activation and upregulation of a functional C5aR, C3aR expression and function were also studied. In analogy to C5a, MC activation by Ag (following IgE priming) or ionomycin increased chemotaxis toward C3a in vitro (Fig. 4A). However, C3a proved to be less efficient (migration optimum at 1000 ng/ml) than C5a (optimum at 100 ng/ml) and less potent (lower numbers of migrated cells) (Fig. 4B). The specificity of C3a-induced chemotaxis was demonstrated by preincubating MC with C3a which abrogated migration most likely as a consequence of C3aR desensitization. The low efficiency and potency of C3a as a MC chemotaxin was confirmed by our finding that C3a failed to mobilize activated MC in vivo (Fig. 4C) but, on the other hand, recruited human and murine macrophages [[18]; J.Z, personal communication]. Furthermore, C3a wa unable to induce intracellular calcium fluxes in activated MC, in contrast to C5a (Fig. 4D). Proving the functional integrity of the C3a preparation used herein, C3a was found to be a potent inducer of chemotaxis and calcium release in J774A.1 macrophages (data not shown).

### SCF-independent C5aR upregulation

**Figure 5:**
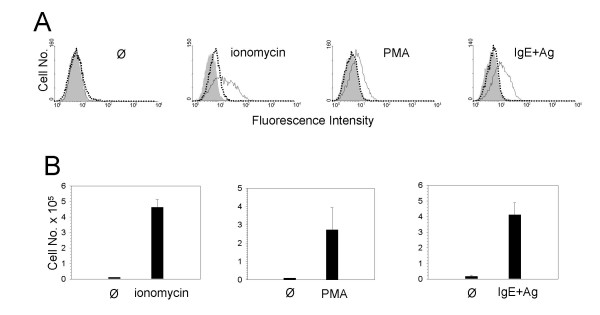
**C5aR upregulation is independent of SCF treatment**. Precursor cell-derived murine MC cultured with IL-3 were treated or not (Ø) with ionomycin, PMA, or Ag (DNP-albumin, following a 24 h preincubation period with IgE anti-DNP) for 24 h (A) or 4 h (B). In (A), MC were stained by indirect immunofluorescence and analyzed by FACS. Filled histograms indicate staining with rat IgG1 control mAb, open histograms (solid lines) with mAb 1240 against murine C5aR, and open histograms (dotted lines) with mAb 1G4 against murine C3aR. In (B), MC were labeled with PKH26 and injected i.v. into syngeneic BALB/c mice together with C5a (10 μg) i.p.. 24 h later, peritoneal cells were harvested and labeled migratory cells identified by FACS analysis. One representative experiment of 3 (A) and mean values (± SEM) of 4 independent experiments each (B) are shown.

To exclude an impact of SCF treatment on C5aR expression, we generated MC in vitro by culturing bone marrow-derived precursor cells in the sole presence of IL-3. Fig. 5A demonstrates that MC cultured in the absence of SCF were also subject to upregulation of surface C5aR by stimulation with ionomycin, PMA, or Ag. C5aR on IL-3-treated MC was functional since they vigorously migrated in vivo in response to C5a (Fig. 5B).

### Anaphylatoxin receptors on peritoneal MC

**Figure 6:**
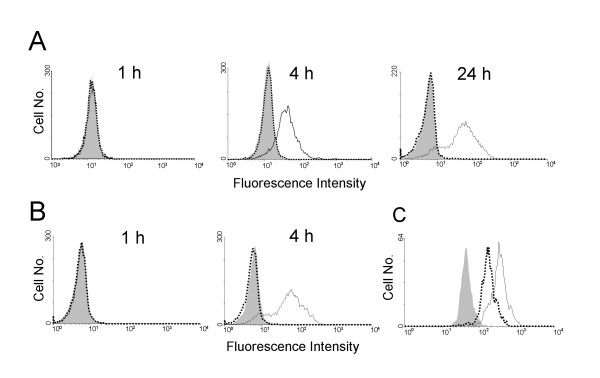
**Activation of peritoneal MC induces C5aR upregulation**. Peritoneal MC from BALB/c mice were purified by a negative selection technique and cultured with ionomycin (A) or Ag (DNP-albumin, following a 24 h preincubation period with IgE anti-DNP) (B) for different periods as indicated. Subsequently, MC were stained by indirect immunofluorescence and analyzed by FACS. Gates were set on CD117^+ ^MC (A, B). In (C), untreated peritoneal lavage cells were stained by indirect immunofluorescence and gates set on F4/80^+ ^macrophages. Filled histograms indicate staining with rat IgG1 control mAb, open histograms (solid lines) with mAb 1240 against murine C5aR, and open histograms (dotted lines) with mAb 1G4 against murine C3aR. One representative experiment each of 3 is shown.

After establishing the correlation between C5aR expression and cellular activation in precursor cell-derived MC, we also investigated primary MC obtained from the peritoneal cavity. Similar to in vitro generated MC, activation of peritoneal MC for 1 h with ionomycin (Fig. 6A) or Ag (following IgE priming) (Fig. 6B) failed to induce C5aR surface expression. After 4 or 24 h of stimulation, however, C5aR expression was extensive whereas C3aR remained undetectable (Fig. 6A, B). C5aR and C3aR were also found to be prominently expressed on resting peritoneal macrophages (Fig. 6C) whereas no other cell type constitutively present in the peritoneal cavity expressed detectable amounts of anaphylatoxin receptors (data not shown).

## Discussion

MC development is a complex process resulting in phenotypically distinct populations at different anatomical sites. In rodents, two MC subsets are discriminated on the basis of different staining characteristics called connective tissue-type MC (CTMC) which are present in the skin and peritoneal cavity, and mucosal MC found in the intestinal or airway mucosa [19,20]. In humans, two potentially analogous MC populations have been defined on the basis of the protease content of their granules with MC_T _containing tryptase and MC_TC _tryptase plus chymase [21].

Murine CTMC from the skin appear to constitutively express C5aR as they were found to degranulate in response to C5a [15]. Likewise, expression of a functional C5aR has been detected on human skin-derived MC_TC _as opposed to MC_T _from lung, kidney and intestine [9-12,22,23]. C5aR was also observed to distinguish the MC_TC _from the MC_T _type of human lung MC [24]. These findings suggest that C5aR is constitutively expressed in murine CTMC as well as in human MC_TC_. However, MC phenotypes are also subject to change in the context of inflammation and infection [25]. For example, synovial MC_TC _expressed C5aR exclusively in inflamed tissues of rheumatoid arthritis patients [13,14] raising the possibility that C5aR expression could also depend on variations in the local microenvironment.

In the present study, precursor cell-derived murine MC were found to migrate towards C5a in vitro, confirming previous data [26,27]. On the other hand, surface C5aR on cultured, resting MC was below the theshold of flow cytometric detection which may explain why C5a failed to induce intracellular calcium fluxes and migration in vivo. However, activated MC were distinguished by a prominent C5aR expression and vigorous functional responsiveness to C5a in vitro as well as in vivo which required approximately 4 h of stimulation by Ag (subsequent to IgE primimg), ionomycin, or PMA. As the signaling cascade downstream of the high affinity receptor for IgE, FcεRI, also results in PKC activation and intracellular calcium fluxes [28], it remains to be shown if other physiologic MC activators may stimulate C5aR upregulation as well.

SCF which leads to the activation of muliple signaling pathways after binding to its receptor induces IL-3-dependent MC to mature and acquire characteristics of CTMC [29,30]. Our study demonstrates, however, that the capacity of precursor cell-derived MC to upregulate a functional C5aR was independent of SCF-induced MC differentiation. This finding could indicate that the activation-induced neoexpression of surface C5aR on MC may not be restricted to CTMC. Further detailed studies are needed to resolve this issue.

Having established the correlation between MC activation and C5aR expression in precursor cell-derived MC, we also studied primary murine MC obtained from the peritoneal cavity which have characteristics of CTMC. Originally, peritoneal MC were described to be unresponsive to C5a [15]. Recently, however, a study utilizing genetically deficient mice suggested C5aR on peritoneal MC to be instrumental in a model of zymosan-induced peritonitis [16]. The evidence presented herein clearly indicates that resting peritoneal MC lack surface C5aR which is in accordance with the original finding of Lim et al. (1991). However, following stimulation with Ag (subsequent to IgE priming) or ionomycin, we found peritoneal MC to acquire an inflammatory phenotype distinguished by a prominent C5aR expression. Thus, one may hypothesize that peritoneal MC gain responsiveness to C5a by C5aR upregulation as a consequence of zymosan-induced inflammation which is additionally characterized by the accumulation of C5a due to the vigorous activation of the complement cascade.

C3a was found to be a potent chemotaxin for HMC-1 mast cells [11,12]. However, this tumor cell line may not be fully representative for primary human MC since tumor transformation may substantially alter normal cell functions. Investigating skin-derived human MC, Hartmann et al. [12] found that C3a chemoattracted only one out of two MC preparations whereas C5a was effective in both. Likewise, El-Lati et al. [8] reported C3a to be a weak inducer of histamin release as compared to C5a. Notably, rat peritoneal MC responded to C3a in a receptor-independent manner [17]. In agreement with these observations, we found murine peritoneal MC to lack surface C3aR, the expression of which, in contrast to C5aR, was not inducible by MC activation. Furthermore, the activation-induced upregulation of C3aR in precursor cell-derived MC was low as compared to C5aR and C3a, in contrast to C5a, was unable to stimulate calcium fluxes in activated MC or mobilize these cells in vivo.

Thus, our findings argue against a significant role for C3aR in MC-mediated pathogenesis. In accordance with this hypothesis, immune complex-induced skin injury and peritonitis were found to be dependent on MC and C5aR, but not C3aR or C3 [31,32]. It is important to note, however, that the results and conclusions of the present study exclusively refer to the mouse.

## Conclusion

Our study demonstrates for the first time that Ag-dependent as well as -independent activation induces an inflammatory MC phenotype which is distinguished by neoexpression of a functional C5aR as a novel effector mechanism in MC-mediated pathogenesis.

## Methods

### Reagents

Recombinant murine IL-3 and SCF were obtained from PeproTech (Cell Concepts, Umkirch, Germany). Recombinant human C5a was described elsewhere [18]. Recombinant human C3a and PMA were from Calbiochem (Merck Biosciences, Darmstadt, Germany). Ionomycin, monoclonal murine IgE anti-DNP, and DNP-albumin were from Sigma-Aldrich (Deisenhofen, Germany).

### Monoclonal Ab against murine C5aR and C3aR

Anti-murine C3aR mAb 1G4 (rat IgG1) has been described by us [33]. Our laboratory has also reported generation of several mAbs against murine C5aR [34]. From that fusion, mAb 1240 was now selected for use in the present study as its isotype (rat IgG1) was identical to mAb 1G4.

### Murine MC preparations

Murine precursor cell-derived MC were generated as described [35,36], with minor modifications. In brief, bone marrow was collected from tibias and femurs of female BALB/c mice, passed through a nylon mesh to remove small pieces of bone and debris, resuspended in complete medium (RPMI 1640 containing 10% FCS, 0.1 mM nonessential aa, 2 mM L-glutamine, 100 U/ml penicillin, 100 μg/ml streptomycin, 1 mM sodium pyruvate, 50 μM 2-ME), and cultured for 2 h. Nonadherent cells were collected, and aliquots of 5 × 10^5 ^cells placed in 24-well plates containing 1 ml of complete medium together with murine IL-3 in the presence or absence of murine SCF (10 ng/ml each). Two-thirds of the medium was replaced every 3–4 days. After 5–10 weeks, more than 95% of nonadherent cells were MC, as judged by morphology, surface expression of CD117 and IgE binding. MC were activated either with ionomycin (750 ng/ml) or PMA (50 ng/ml) for different periods as indicated or, in an Ag-specific manner, by preincubation with monoclonal murine IgE anti-DNP (2 μg/ml) for 24 h followed by the Ag DNP-albumin (50 μg/ml) for different periods as indicated.

Peritoneal MC were purified from lavage cells of BALB/c mice by magnetic cell sorting using a negative selection technique to avoid MC activation. For this purpose, approximately 1 × 10^8 ^peritoneal cells were incubated with FITC-anti-CD90 (clone 30H12) and FITC-anti-CD19 (clone 6D5) and T- and B-cells deleted by incubation with anti-FITC immunomagnetic microbeads and high-gradient LS separation columns. In a second step, remaining cells were incubated with FITC-anti-CD11b (clone M1/70), FITC-anti-CD11c (clone N418), FITC-anti-Gr1 (clone RB6-8C5), and FITC-anti-CD49b (clone DX5). Subsequently, monocytes, DC, granulocytes, and NK cells, respectively, were deleted using anti-FITC immunomagnetic microbeads and high-gradient LS separation columns. All mAb and reagents were obtained from Miltenyi Biotec (Bergisch-Gladbach, Germany). Following this procedure, 5 × 10^5 ^to 8 × 10^5 ^cells were recovered which were >80% MC as judged by CD117 surface expression. Purified peritoneal MC were either immediately used for staining and FACS analysis or cultured in complete medium as indicated.

### In vitro chemotaxis

In vitro chemotaxis was assayed using the HTS Transwell-24 system from Corning (Beyer Lab., Düsseldorf, Germany). Cells diluted at 1 × 10^6^/ml in migration buffer (RPMI 1640 with 1% BSA) were placed in the upper wells whereas anaphylatoxins diluted in migration buffer as indicated were added to the lower wells. Polycarbonate membranes with a pore size of 5 μm were used and incubation was performed at 37°C in a 5% CO_2 _atmosphere for 3 h. Migration was stopped and migrated cells detached by placing Transwell chambers for 15 min on ice. Subsequently, migrated cells in the lower chambers were counted using a hemocytometer. All determinations were performed in duplicate.

For desensitization studies, cells (1 × 10^6 ^in 1 ml medium) were preincubated at 37°C for 30 min with the indicated anaphylatoxin (200 ng/ml) and centrifuged.

### In vivo migration model

All animal work was conducted in accordance with guidelines for animal welfare and was approved by the government of Lower Saxony, Germany. In vivo migration was studied as described elsewhere [18]. Murine bone marrow-derived MC were labeled with the red fluorescent dye PKH-26 (Sigma-Aldrich) according to the manufacturer's instructions. Cells (1 × 10^7 ^in 200 μl PBS) were then injected into the tail vein of BALB/c mice (weight 20–24 g; age 8–20 wk) together with a chemotaxin (10 μg C5a or 50 μg C3a; in 200 μl PBS) which was injected into the peritoneal cavity. Approximately 24 h later, mice were sacrificed and peritoneal lavage performed. Subsequently, peritoneal cells were counted and analyzed by FACS. Absolute numbers of migrated labeled cells were calculated from the percentage of red fluorescent cells as determined by FACS analysis and the total peritoneal cell count.

For C5aR blockade, labeled MC (1 × 10^7 ^in 1 ml PBS) were preincubated on ice for 45 min with 20 μg of mAb 1240 (or, as a control, mAb 1G4; both are rat IgG1), washed twice with PBS and resuspended in 200 μl PBS for injection.

For desensitization studies, labeled MC (1 × 10^7 ^in 1 ml PBS) were preincubated at 37°C for 1 h with 2 μg of anaphylatoxin as indicated, washed twice in PBS and resuspended in 200 μl PBS for injection.

### Calcium measurements

A total of 10^6 ^cells were loaded in 700 μl RPMI containing 5% FCS, 1 μM Fluo3-AM and 0.02% Pluronic F127 (both from Molecular Probes, Invitrogen, Karlsruhe, Germany). Subsequently, the cell suspension was diluted 2-fold with RPMI 10% FCS and was incubated for 10 min at 37°C. Cells were washed twice with Krebs Ringer solution composed of 10 mM HEPES (pH 7.0), 140 mM NaCl, 4 mM KCl, 1 mM MgCl_2_, 1 mM CaCl_2_, and 10 mM glucose. The changes in fluorescence intensity of Fluo3 were monitored on a LSRII cytometer (BD Biosciences, Heidelberg, Germany). Loading of the samples was controlled by treatment with 750 ng/ml ionomycin.

### FACS analysis

For indirect immunofluorescence analysis, MC (2 × 10^5 ^in 100 μl) were washed with PBS containing 1.5% FCS and 0.1% NaN_3 _and blocked with heat aggregated human IgG (20 μg) for 20 min on ice. After a washing step, primary mAb in PBS containing 1.5% FCS, heat aggregated human IgG (10 μg in 100 μl) and 0.1% NaN_3 _were added and incubated for 45 min on ice. After washing cells three times in PBS/1.5% FCS/0.1% NaN_3_, biotin-conjugated mouse anti-rat IgG1 (BD Biosciences) was added for another 45 min. After washing cells three times in PBS/1.5% FCS/0.1% NaN_3_, streptavidin-FITC (Dako, Hamburg, Germany) was added for another 45 min. If peritoneal cells were studied, PE-anti-murine CD117 (rat IgG2b; Miltenyi Biotec) or PE-anti-F4/80 (Caltag Lab., Hamburg, Germany) were included in this step to identify MC or macrophages. Finally, cells were washed three times in PBS/1.5% FCS/0.1% NaN_3_, resuspended in PBS containing 1% formaldehyde and analyzed by flow cytometry (FACSCalibur; BD Biosciences). If peritoneal cells were studied, gates were set on CD117+ MC or F4/80+ macrophages.

The following primary mAb were used at a concentration of 5 μg/ml: anti-murine C5aR mAb 1240 (rat IgG1), anti-murine C3aR mAb 1G4 (rat IgG1), isotype control mAb rat IgG1 (BD Biosciences).

## Abbreviations

MC: mast cell; CTMC: connective tissue-type MC; C5aR: C5a receptor; C3aR: C3a receptor.

## Authors' contributions

AS, JG, ZK and JZ were all involved in the study design, data acquisition, analysis and interpretation. JZ drafted the manuscript. All authors read and approved the final manuscript.
